# Reconfigurable Optoelectronic Logic Gates Enabled by a VSe_2_/MoTe_2_ van der Waals Heterojunction

**DOI:** 10.3390/nano16191247

**Published:** 2026-10-02

**Authors:** Kang Zhang, Wansong He, Ruya Cong, Xin Zhang, Jianzhi Gao, Yubing Tu, Suofu Wang, Mingsheng Long

**Affiliations:** 1 State Key Laboratory of Opto-Electronic Information Acquisition and Protection Technology, Institutes of Physical Science and Information Technology, Anhui University, Hefei 230601, China; 2 School of Physics and Information Technology, Shaanxi Normal University, Xi’an 710119, China; 3 Key Laboratory of Opto-Electronic Information Acquisition and Manipulation of Ministry of Education, Anhui University, Hefei 230601, China; 4 School of Energy, Power and Safety, Anhui University of Applied Technology, Hefei 230011, China

**Keywords:** optoelectronic logic gates, van der Waals heterojunction

## Abstract

Optoelectronic logic gates that integrate optical inputs with electrical outputs offer a promising route toward high-bandwidth, low-power in-sensor computing. However, most existing implementations require precise control over the excitation wavelength, optical power, or gate bias, which complicates device fabrication and system integration. Here, we demonstrate a reconfigurable optoelectronic logic device based on a VSe_2_/MoTe_2_ van der Waals heterojunction. The device exhibits a rectification ratio exceeding 10^3^ in the dark and a pronounced photovoltaic response, including a light on/off ratio surpassing 10^7^, a responsivity of 0.28 A/W, and external quantum efficiency of 54%, as well as a noise-equivalent power of 4.6 × 10^−14^ W/Hz^1/2^ and a specific detectivity of 2.1 × 10^10^ cm Hz^1/2^/W. By reconfiguring the source and drain electrodes and modulating the optical input, six fundamental Boolean logic operations—AND, IMP, NOR, NOT, OR, and NAND—are realized in the device. Notably, all six logic functions are obtained under fixed illumination and predefined bias, without requiring wavelength, power, or gate modulation. Switching between functions requires only a specific bias voltage and a change in the source/drain connection (for certain operations, OR and NAND, the input state is redefined). These results underscore the promise of two-dimensional material-based van der Waals heterojunctions for compact, low-power, and reconfigurable optoelectronic logic processing.

## 1. Introduction

Logic circuits constitute the fundamental building blocks of modern computing and information processing, with widespread applications ranging from artificial intelligence and data communication to intelligent sensing [1,2]. In conventional electronic logic circuits, Boolean operations are executed through interconnected transistors, in which the output logic states are determined by voltage or current levels [3,4]. However, the exponential growth of data traffic, coupled with the ever-increasing demand for low power consumption, has imposed critical bottlenecks, including resistance–capacitance delays, high energy dissipation, and electromagnetic interference [5]. Optoelectronic logic gates, which directly convert optical inputs into electrical outputs, offer a compelling alternative by combining the high bandwidth of optical transmission with the convenience of electrical readout, thereby facilitating in-sensor computing with reduced latency and energy consumption [6,7].

Most existing optoelectronic logic gates that support multiple Boolean operations require careful selection of the excitation wavelength and optical power to achieve different logic functions. They also tend to rely on additional bias modulation and complex device architectures. Collectively, these requirements significantly complicate device fabrication and system integration [8,9]. To address these challenges, bipolar photoresponse—in which positive and negative photocurrents coexist in a single device—has been exploited as a promising route [10,11]. For example, wavelength-dependent bipolar photoresponse has been demonstrated in heterostructures such as b-AsP/MoTe_2_ and GaSe/GaN, enabling multiple logic operations [12,13]. Gate-voltage modulation has also been widely adopted to switch photocurrent polarity in field-effect transistors [14,15]. In addition, ferroelectric polarization switching has been utilized to achieve bipolar photocurrents via photoinduced polarization reversal [16], and perovskite-based photodetectors as well as graphdiyne/graphene heterostructures have also been explored for multifunctional logic operations [9,17]. Among these platforms, Schottky junctions based on two-dimensional (2D) materials have attracted particular interest, offering atomically sharp interfaces and reduced Fermi-level pinning compared with conventional metal–semiconductor contacts [18,19]. By using 2D metals or semimetals as contact electrodes, clean heterointerfaces can be formed, enabling flexible tuning of band alignment [20,21,22]. Gate-tunable rectification and polarity conversion have been demonstrated in a 1T-VSe_2_/2H-MoTe_2_ Schottky junction, where an additional gate terminal is required to modulate the built-in electric field direction and thereby achieve near-infrared switchable logic [23]. Reconfigurable polarity in WSe_2_ field-effect transistors has also been achieved through dual-gate control [24]. Despite recent progress, the reliance on wavelength-selective excitation, gate-voltage modulation, and optical power modulation remains unresolved. This motivates the search for a simpler and better-defined strategy based on alternative materials or heterojunctions to achieve a multifunctional optoelectronic logic gate.

In this work, we demonstrate an optoelectronic logic device based on a VSe_2_/MoTe_2_ heterostructure. The device exhibits a rectification ratio exceeding 10^3^ under dark conditions and a robust self-powered photoresponse, with an ultrahigh light-to-dark current ratio surpassing 10^7^ at 0 V bias. In the photovoltaic mode, a high responsivity of 0.28 A/W and an EQE of 54% are obtained, with a corresponding noise equivalent power of 4.6 × 10^−14^ W/Hz^1/2^ and a specific detectivity of 2.1 × 10^10^ cm Hz^1/2^/W. Exploiting the reconfigurable source–drain configurations, we realize six fundamental Boolean logic operations—AND, IMP, NOR, NOT, OR, and NAND—within a device through modulated optical inputs. Logic functions are selected via the specific bias voltage and source/drain connection, with the input state redefined only for OR and NAND. Our results provide a compact, low-power platform for on-chip optoelectronic logic processing, with promising potential for intelligent sensing and optical communication systems.

## 2. Materials and Methods

Single crystals of VSe_2_ and MoTe_2_ were grown by the chemical vapor transport method [25,26]. Single-crystal X-ray diffraction (XRD) patterns were collected using a Rigaku SmartLab diffractometer (Rigaku Corp., Tokyo, Japan) with Cu-Kα1 radiation at room temperature. The samples were characterized using an atomic force microscope (AFM, Oxford, Santa Barbara, CA, USA). Raman spectra were measured with a 532 nm laser as the excitation source (Renishaw, InVia-Reflex, Renishaw plc, Wotton-under-Edge, UK).

VSe_2_ and MoTe_2_ nanoflakes were prepared by the standard mechanical exfoliation method. The VSe_2_/MoTe_2_ heterojunction device was fabricated using dry transfer technology. The top metal electrodes were patterned through standard electron beam lithography (EBL) and thermal evaporation of Cr/Au electrodes (typically 10 nm Cr and 80 nm Au). Electrical properties and photoresponse tests were conducted using a dual-channel digital source meter (Keithley 2636B, Keithley Instruments, Cleveland, OH, USA). The 637 nm excitation laser was focused onto the device through a 20× objective lens. All measurements were performed in ambient air at room temperature.

## 3. Results and Discussion

**Figure 1:**
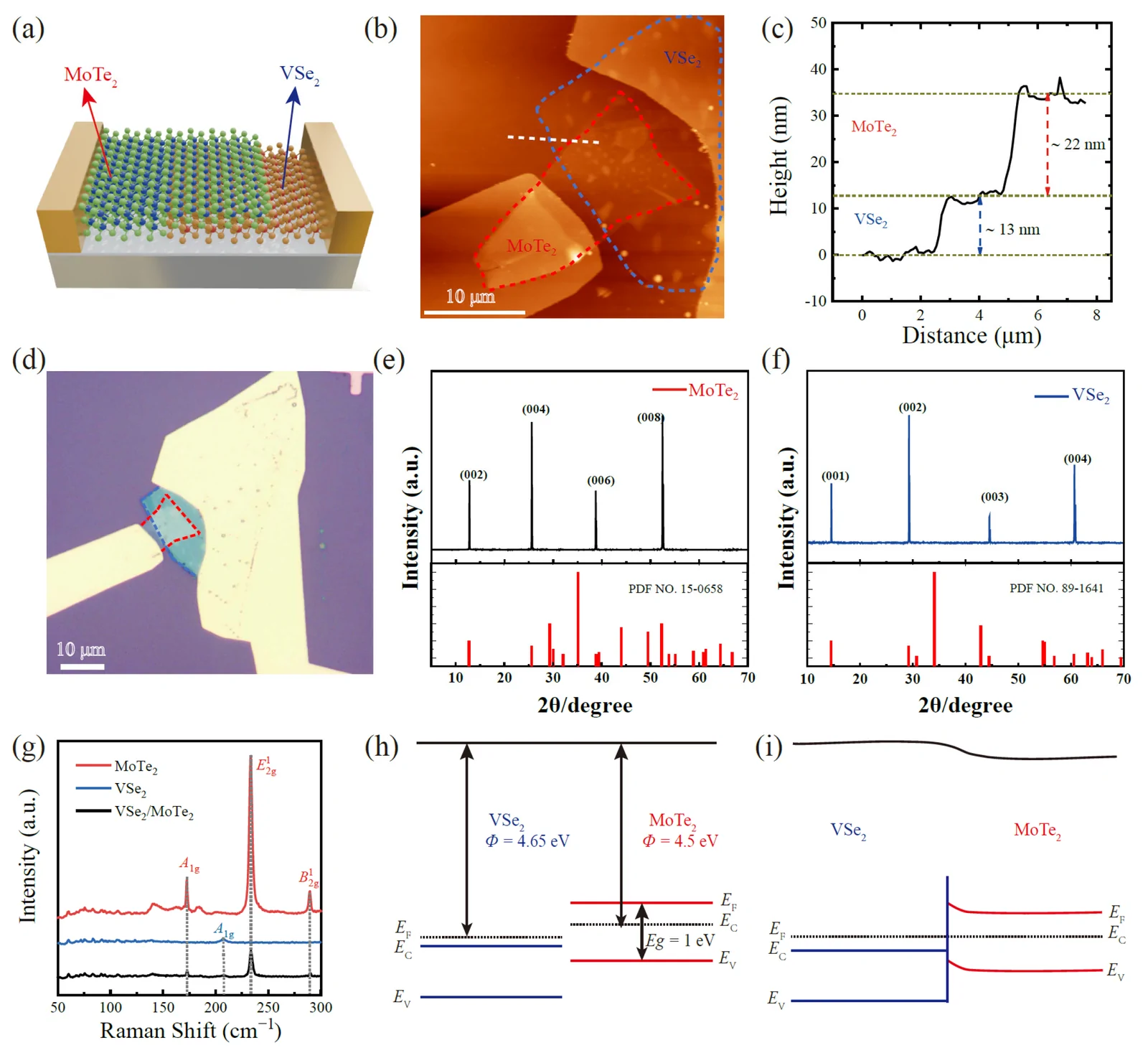
Characterization and energy band alignment of the VSe_2_/MoTe_2_ heterostructure device. (**a**) Schematic diagram of the VSe_2_/MoTe_2_ heterostructure device. (**b**) AFM image of the VSe_2_/MoTe_2_ heterostructure. Red and blue dashed lines show multilayer MoTe_2_ and VSe_2_, respectively. (**c**) Corresponding height profile along the white dashed line in (**b**). (**d**) Optical image of the VSe_2_/MoTe_2_ heterostructure device. Red and blue dashed lines show multilayer MoTe_2_ and VSe_2_, respectively. (**e**,**f**) XRD patterns of MoTe_2_ (**e**) and VSe_2_ (**f**), respectively. (**g**) Raman spectra of VSe_2_, MoTe_2_, and the VSe_2_/MoTe_2_ heterostructure. (**h**,**i**) Energy band diagrams of VSe_2_ and MoTe_2_ before (**h**) and after (**i**) contact.

The three-dimensional structural schematic of the VSe_2_/MoTe_2_ heterojunction is shown in Figure 1a, with the vertical stacking sequence from top to bottom being MoTe_2_ and VSe_2_, respectively. Single crystals of VSe_2_ and MoTe_2_ were verified by X-ray diffraction, (Figure 1e,f). Mechanical exfoliation of the bulk crystals yielded thin flakes of MoTe_2_ and VSe_2_, which were then integrated into the heterostructure device via dry transfer and thermal evaporation. An optical microscopy image of the device is shown in Figure 1d. Atomic force microscopy measurements revealed thicknesses of ~13 nm for VSe_2_ and ~22 nm for MoTe_2_ (Figure 1c), with the red and blue regions in Figure 1b denoting the respective flakes. Raman spectroscopy further confirmed the heterostructure formation, as shown in Figure 1g. The Raman spectrum exhibits characteristic peaks at 170, 234, and 289 cm^−1^, which are assigned to the *A*_1__g_, $E_{2g}^{1}$, and $B_{2g}^{1}$ modes of 2H-phase MoTe_2_ [27]. An additional faint peak observed at 206 cm^−1^ corresponds to the *A*^1^_g_ mode of 1T-phase VSe_2_ [28]. All the characteristic peaks of both VSe_2_ and MoTe_2_ were identified in the overlapping region of the two materials, confirming the successful formation of a high-quality van der Waals heterostructure.

MoTe_2_ exhibits a conduction band minimum of −3.8 eV, a valence band maximum of −4.8 eV, and a band gap of ~1.0 eV [29]. The work function of VSe_2_ has been reported to be approximately 4.65 eV, yielding a work function difference (Δ*E*_F_) of ~150 meV relative to MoTe_2_ [23]. The energy band alignment diagrams of MoTe_2_ and VSe_2_ before and after contact are illustrated in Figure 1h,i, respectively. Owing to the higher Fermi level of MoTe_2_ relative to that of VSe_2_, electrons diffuse from MoTe_2_ to VSe_2_ upon contact. Consequently, a built-in electric field is established at the interface, directed from MoTe_2_ to VSe_2_, accompanied by slight band bending at the interface, as shown in Figure 1i. This built-in field facilitates the separation and transport of photogenerated carriers.

**Figure 2:**
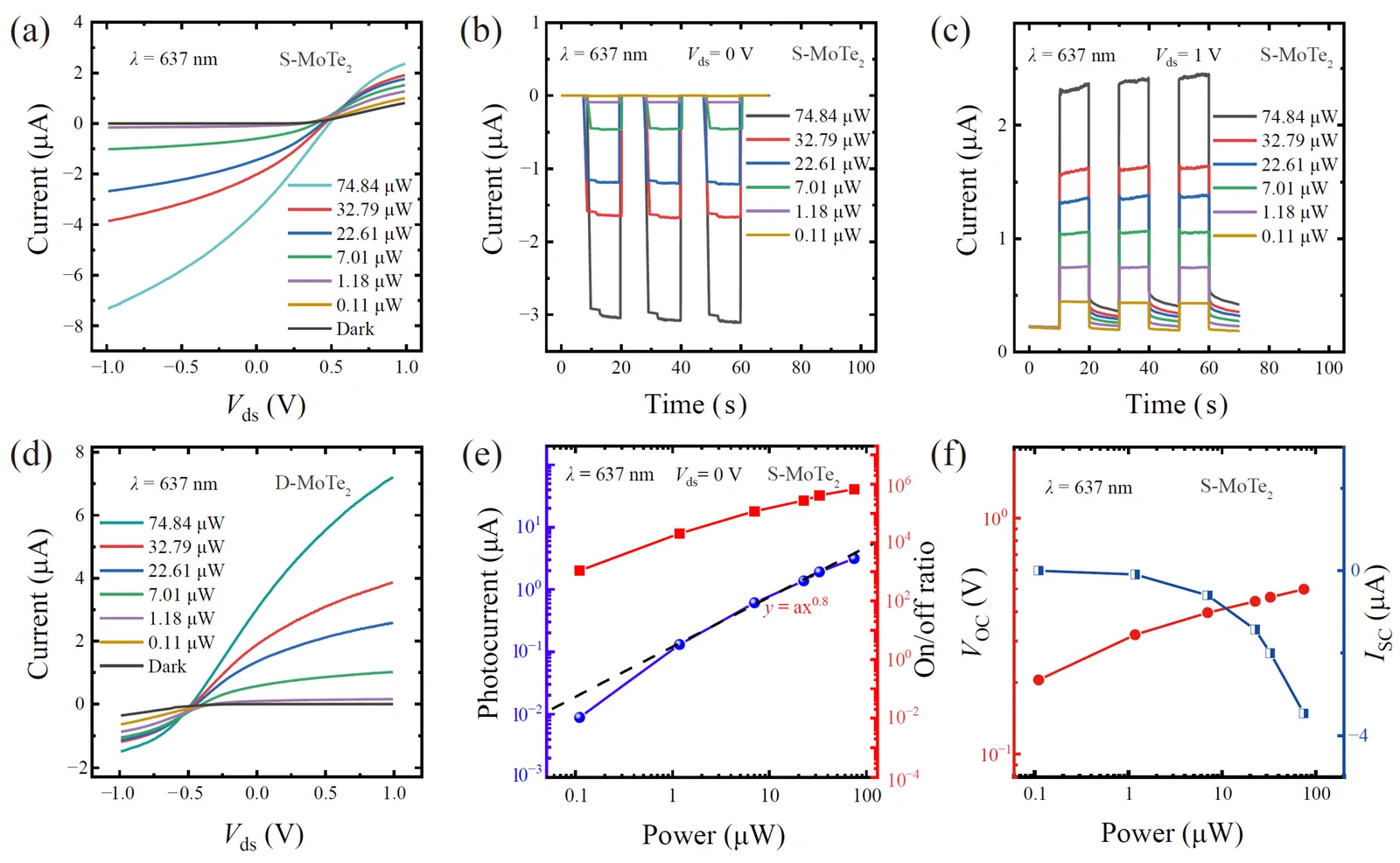
Photoresponse characteristics of the VSe_2_/MoTe_2_ heterostructure device under 637 nm laser illumination. (**a**) *I*-*V* curves of the S-MoTe_2_ configuration under different illumination powers. (**b**,**c**) Time-resolved photoresponse of the S-MoTe_2_ configuration under different illumination powers at *V*_ds_ = 0 and +1 V, respectively. (**d**) *I*-*V* curves of the D-MoTe_2_ configuration under different illumination powers. (**e**) Extracted photocurrent and calculated on/off ratio as a function of illumination power for the S-MoTe_2_ configuration at *V*_ds_ = 0 V. (**f**) Open-circuit voltage and short-circuit current as a function of illumination power for the S-MoTe_2_ configuration.

The photoelectric performance of the VSe_2_/MoTe_2_ heterostructure under 637 nm laser illumination was systematically investigated for different electrode connection configurations. For the case where MoTe_2_ is connected to the source electrode (S-MoTe_2_), Figure 2a presents the current–voltage (*I*-*V*) characteristic curves of the VSe_2_/MoTe_2_ device. The clear rectification in the dark confirms the formation of a Schottky barrier at the VSe_2_/MoTe_2_ interface. Upon illumination, the built-in field in the depletion layer separates photogenerated electron–hole pairs, shifting the *I*-*V* curve downward and producing a distinct open-circuit voltage (*V*_oc_). The increased photocurrent under negative bias further demonstrates enhanced carrier extraction, consistent with the efficient separation of photogenerated carriers by the built-in field.

The temporal photoresponse of the VSe_2_/MoTe_2_ heterostructure was investigated under various incident light powers and bias voltages (*V*_ds_). As plotted in Figure 2b and Figure 2c, the device exhibits negative and positive photoconductance at 0 V and +1 V, respectively. The dependence of the photocurrent (*I*_ph_), the on/off ratio, *V*_OC_, and the short-circuit current (*I*_SC_) on light power is presented in Figure 2e,f. Here, *I*_ph_ is defined as *I*_ph_ = *I*_light_ − *I*_dark_, where *I*_light_ and *I*_dark_ represent the currents with and without illumination, respectively. The photocurrent exhibits a monotonic increase with increasing light power. As the light power increases from 0.11 to 74.84 μW, the photocurrent increases from 0.01 to 3.13 μA at 0 V. Meanwhile, the light power dependence of the photocurrent was fitted using a power-law relationship *I*_ph_ ∝ P^α^, yielding α of approximately 0.8, which indicates a sublinear response commonly attributed to trap states [21]. Benefiting from the Schottky barrier, the device exhibits an excellent light on/off ratio of over 10^7^ at 0 V, attesting to its pronounced photovoltaic response. Under an illumination power of 74.84 μW, a *V*_OC_ of +0.5 V and an *I*_SC_ of −4 μA were obtained. These results compare favorably with those of most 2D material-based photovoltaic devices [30], demonstrating that the VSe_2_/MoTe_2_ heterostructure device is a highly competitive photovoltaic candidate.

When the source and drain electrodes are swapped, the *I*-*V* characteristics of the VSe_2_/MoTe_2_ device are as shown in Figure 2d. These curves, along with the sign of *V_OC_*, exhibit a centrosymmetric reversal (i.e., a 180°clockwise rotation). This behavior is expected because the built-in electric field remains unchanged, whereas the polarities of both the applied bias and the measured current are reversed. We further investigated the photoresponse for the configuration with MoTe_2_ connected to the drain electrode (D-MoTe_2_), as shown in Figure S1. It can be seen that the photocurrent, on/off ratio, *V*_OC_ and *I*_SC_ are comparable to those of the S-MoTe_2_ case at 0 V. The temporal photoresponse under different bias voltages was also measured, as presented in Figures S2 and S3. It is evident that the device can exhibit opposite photoresponses over a wide bias range, which is essential for realizing reconfigurable optoelectronic logic gates, as will be discussed later.

**Figure 3:**
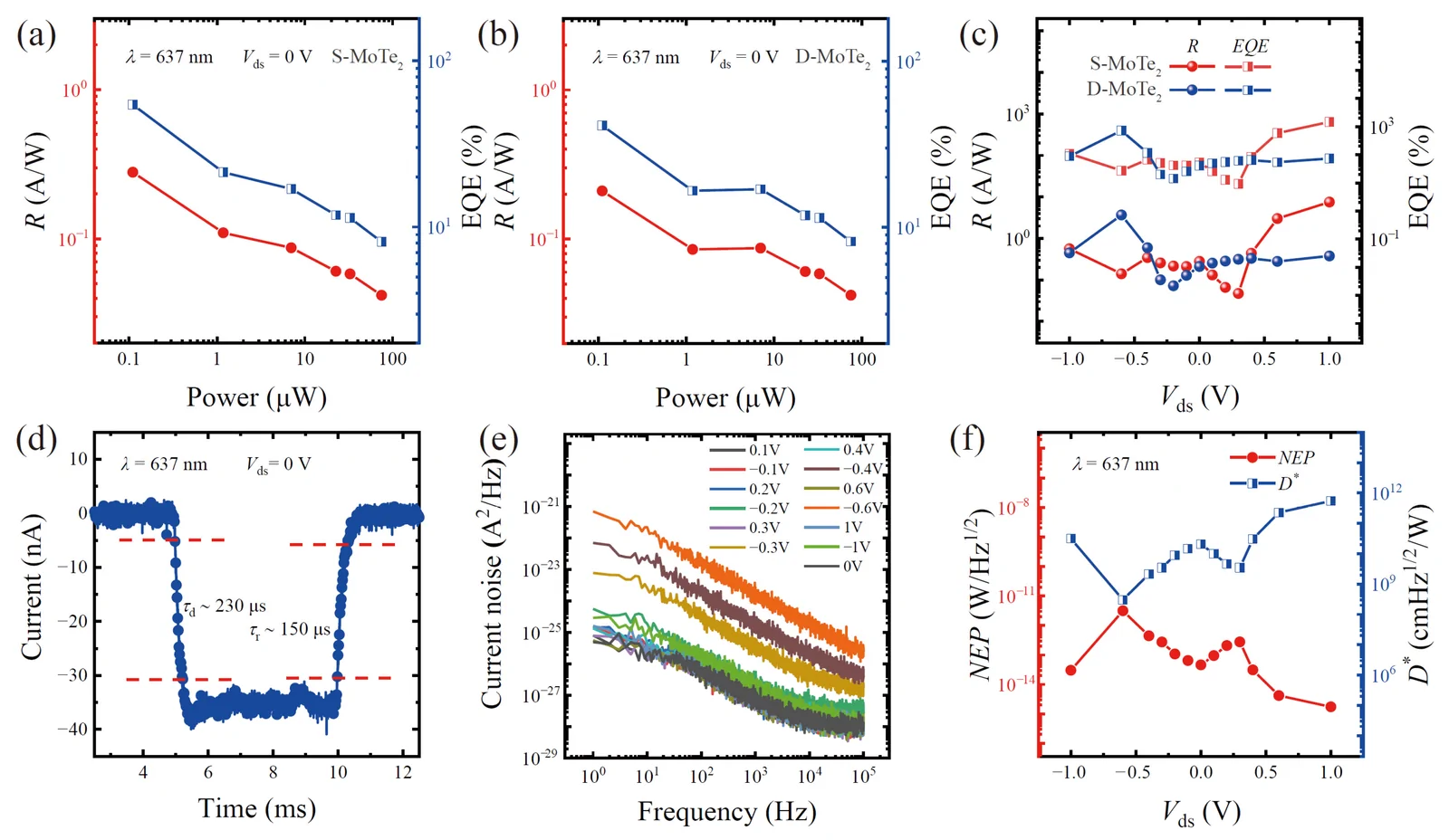
Photodetection performance of the VSe_2_/MoTe_2_ heterostructure device under 637 nm laser illumination. (**a**,**b**) Calculated *R* and EQE as a function of illumination power for the S-MoTe_2_ (**a**) and D-MoTe_2_ (**b**) configurations at *V*_ds_ = 0 V, respectively. (**c**) *R* and EQE as a function of *V*_ds_ for the S-MoTe_2_ and D-MoTe_2_ configurations. (**d**) Temporal photoresponse and extracted rise/decay times of the device at *V*_ds_ = 0 V. (**e**) Current-noise spectral density at different bias voltages. (**f**) Calculated *NEP* and D* as a function of *V*_ds_ for the S-MoTe_2_ configuration.

To evaluate the photoresponse performance of the device, the responsivity (*R*) and external quantum efficiency (EQE) of the VSe_2_/MoTe_2_ heterostructure were further quantified under various bias voltages for different source/drain connections. These parameters were calculated using the expressions *R* = *I*_ph_/*P*_I_ and EQE = (*hcR*)/(*eλ*), where *P*_I_ is the incident light power, and *h*, *c*, *e*, and *λ* are the Planck constant, the speed of light, the elementary charge, and the wavelength, respectively [31]. Figure 3a shows the *R* and corresponding EQE values under different illumination powers at 0 V for the S-MoTe_2_ case. Both *R* and EQE decrease with increasing incident power, which can be attributed to trap states [32]. An *R* of 0.28 A/W and an EQE of 54% were obtained at a light power of 0.11 μW. The *R* and EQE under different illumination powers for the D-MoTe_2_ case are presented in Figure 3b, and their behavior is almost identical to that observed in the S-MoTe_2_ case. In addition to 0 V, the dependence of *R* and EQE on light power at additional bias voltages is plotted in Figure S4. As summarized in Figure 3c, the bias-dependent *R* and EQE exhibit a centrosymmetric reversal between the S-MoTe_2_ and D-MoTe_2_ configurations, consistent with that observed in the *I*-*V* curves of the two configurations, which can be attributed to the pronounced photovoltaic effect. These results further demonstrate that the device exhibits good photoresponse performance in both configurations.

Next, the speed of the photoresponse, one of the most important figures of merit for a photodetector, was measured. The rise time *τ*_r_ and decay time *τ*_d_ were extracted from the 10–90% and 90–10% photocurrent transition intervals, respectively. Under 0 V bias, the VSe_2_/MoTe_2_ heterostructure device exhibited rapid switching with *τ*_r_ = 150 μs and *τ*_d_ = 230 μs, as shown in Figure 3d. Additionally, the noise equivalent power (NEP) and specific detectivity (D*) of the VSe_2_/MoTe_2_ heterostructure were also investigated. NEP represents the ability of the detector to distinguish the light signal from the background noise, while D* reflects the detection limit of the photodetector. NEP was derived from NEP = <*i_n_*^2^>^1/2^/*R*, where *i*_n_ denotes the noise current density, ${\langle {i_{n}}^{2}\rangle }^{1/2}=1/B\int_{0}^{B}S(f)df$ [31]. Figure 3e shows the measured noise spectral density of the VSe_2_/MoTe_2_ device under different biases, which includes frequency-dependent 1/*f* noise and frequency-independent white noise. The specific detectivity D* was calculated using the formula D* = (*S*·Δ*f*) ^1/2^/NEP, where S represents the effective area of the device, and Δ*f* is the electrical bandwidth. The extracted NEP and D* values as a function of bias are shown in Figure 3f. An NEP of 4.6 × 10^−14^ W/Hz^1/2^ and D* of 2.1 × 10^10^ cm Hz^1/2^/W were obtained at 0 V. Over the full bias range, NEP remained below 3.1 × 10^−12^ W/Hz^1/2^, with the corresponding D* above 3 × 10^8^ cm Hz^1/2^/W. Given the fast response time and low noise level, it is promising to construct an optoelectronic logic device based on such a photovoltaic VSe_2_/MoTe_2_ heterostructure.

**Figure 4:**
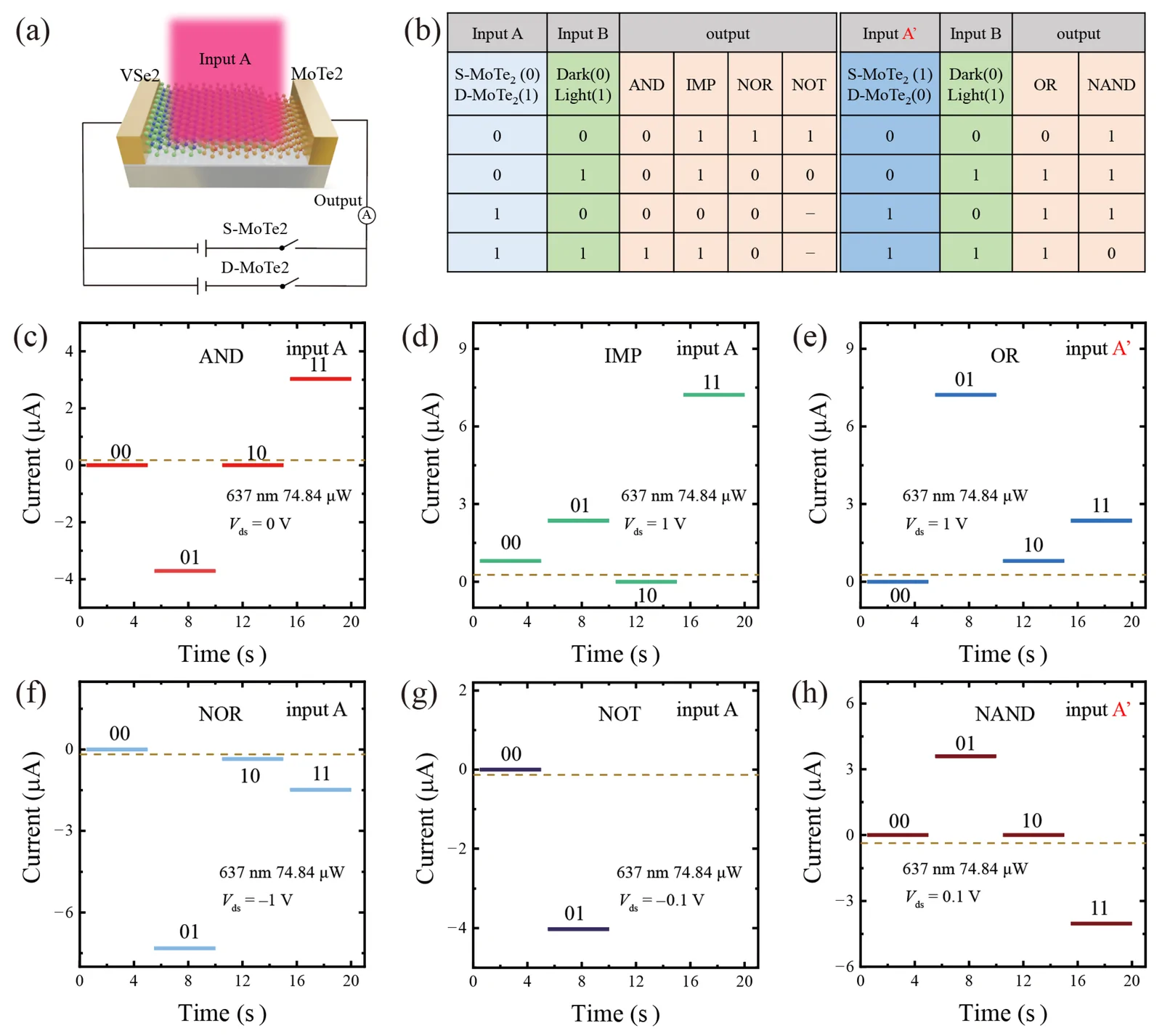
Demonstration of multifunctional optoelectronic logic gates based on the VSe_2_/MoTe_2_ heterostructure device. (**a**) Schematic diagram of the VSe_2_/MoTe_2_ heterostructure device for optoelectronic logic operations. (**b**) Truth table of the AND, IMP, NOR, NOT, OR, and NAND logic gates. (**c**–**h**) Output current curves for the AND, IMP, NOR, NOT, OR, and NAND logic gate operations. The input conditions are marked in (**c**–**h**). The dashed line in (**c**–**h**) represents the fiducial level, which set the output state as “1” and “0” for the values above and below it, respectively.

Inspired by logic operations realized via bipolar photoresponse [7,8,9,10,11,12,13,14,15,33], we implement six Boolean functions in a VSe_2_/MoTe_2_ heterojunction under a fixed illumination condition, as depicted in Figure 4a. In this configuration, the illumination state and the electrode connection serve as the two logic inputs, while the resulting current is read as the logic output. Specifically, input A is defined by the electrode configuration, where “0” and “1” correspond to MoTe_2_ connected to the source and drain electrodes, respectively. Input B is defined by the light states, where “0” and “1” signify light off and on, respectively. Under these definitions, six logical functions—AND, NAND, IMP, NOR, OR, and NOT—can be realized, as listed in Figure 4b.

In detail, only when MoTe_2_ is connected to the drain electrode (input A: “1”) and light is applied (input B: “1”) does the current become distinctly greater than zero, corresponding to the output state of “1” (Figure 4c). Likewise, IMP, NOR, and NOT operations can be realized at different bias voltages, as shown in Figure 4d, Figure 4f and Figure 4g, respectively. Alternatively, the first input can be redefined such that “1” and “0” correspond to MoTe_2_ connected to the source and drain electrodes, respectively (right panel of Figure 4b). Under this definition (input A’), OR and NAND can be realized at 1 V and 0.1 V, respectively, as shown in Figure 4e,h.

Two points should be emphasized in this optoelectronic logic device. First, six logical functions are realized under identical illumination conditions: a fixed wavelength of 637 nm and a fixed optical power of 74.84 μW. This scheme removes the requirement for precisely tuning the wavelength or intensity of the incident light to switch between different logic functions, thereby significantly simplifying the operation. Second, each logic function is accessed at a single, predefined bias voltage. This configuration is straightforward to understand and operate, avoiding the repeated and cumbersome bias adjustment required to obtain a specific logic output. Table 1 summarizes the comparison with previously reported VSe_2_/MoTe_2_ devices that rely on gate modulation [23] and other representative optoelectronic logic devices [7,8,9,13]. Our gate-free architecture simplifies device geometry and operation while maintaining competitive responsivity and response speed under fixed illumination conditions.

## 4. Conclusions

This work demonstrates a gate-free optoelectronic logic device based on the VSe_2_/MoTe_2_ heterojunction, in which six Boolean operations are selected by the source/drain connection under a fixed illumination condition. The heterojunction exhibits pronounced photovoltaic response, with a dark rectification ratio exceeding 10^3^ and light on/off ratio surpassing 10^7^. Meanwhile, the source and drain electrode connections effectively reverse the orientation of the built-in electric field with respect to the external circuit, leading to reversible rectification behavior. Through the source/drain connection configurations and optical illumination, multiple logic functions—including AND, IMP, NOR, NOT, OR, and NAND—are realized within the device. These results highlight a simple operational route toward multifunctional optoelectronic logic in 2D van der Waals heterojunctions.

## Figures and Tables

**Table 1:** Comparison of representative optoelectronic logic devices.

Device Architecture	No. of Gates	Reconfiguration Variable	Illumination Condition	I_ON(logic “1” current)_ −I_OFF(logic “0” current)_	Response Time	External Circuitry	Ref.
2D vertical FET	6	Gate voltage	Varied	~10 nA	—	Required	[7]
vdW junction	7	Gate/junction	Varied	~100 nA	22/14 μs@650 nm 44/20 μs@1550 nm	Required	[8]
Perovskite array	5	Light intensity	Varied	~100 nA	62/680 μs@530 nm 40/72 μs@940 nm	8 × 8 array	[9]
GaSe/GaN Hybrid junctions	7	Wavelength	Varied	~10 nA	180/270 μs@UV 12.5/15 ms@IR 5/7.5 ms@visible	Not required	[13]
VSe_2_/MoTe_2_ vdW junction	4	Gate voltage	Near-infrared	~1 nA	8/8 ms@1064 nm	Required	[23]
VSe_2_/MoTe_2_ vdW junction	6	Source/drain connection	Fixed 637 nm, fixed power	~2 μA	150/230 μs@637 nm	Not required	This work

## Data Availability

The original contributions presented in this study are included in the article/Supplementary Materials. Further inquiries can be directed to the corresponding authors.

## Supplementary Materials

The following supporting information can be downloaded at: https://www.mdpi.com/article/10.3390/nano16191247/s1, Figure S1. Photovoltaic characteristics of the VSe_2_/MoTe_2_ heterostructure device under 637 nm laser illumination. (a) I-V curves of the S-MoTe2 configuration under different illumination powers. (b,c) Time-resolved photoresponse of the D-MoTe2 configuration under different illumination powers at Vds = 0 and −1 V, respectively. (d) I-V curves of the D-MoTe2 configuration under different illumination powers. (e) On/off ratio as a function of illumination power for the D-MoTe2 configuration at Vds = 0 V. (f) Extracted VOC and ISC as a function of illumination power for the D-MoTe2 configuration; Figure S2. Photoresponse characteristics of the VSe_2_/MoTe_2_ heterostructure device at different bias voltages. (a–d) Time-resolved photoresponse of the S-MoTe2 configuration under different illumination powers at 0.6V, −0.6V, −1V, and −0.1V, respectively. (e–h) Time-resolved photoresponse of the D-MoTe2 configuration under different illumination powers at 0.6V, −0.6V, 1V, and −0.1V, respectively. (i) Photocurrent as a function of illumination power for the D-MoTe2 configuration, with the black solid line showing the fit y = ax^0.83^; Figure S3. Bias-dependent photoresponse of the VSe_2_/MoTe_2_ heterostructure device under 637 nm laser illumination. (a,b) Time-resolved photoresponse of the S–MoTe_2_ and D–MoTe_2_ configurations, respectively, under different illumination powers and bias voltages; Figure S4. Power-dependent photodetection performance of the VSe_2_/MoTe_2_ heterostructure device. (a) and (b) R and EQE as a function of illumination power for the S-MoTe2 configuration at different bias voltages, respectively. (c) and (d) R and EQE as a function of illumination power for the D-MoTe2 configuration at different bias voltages, respectively.
