# Supplementary material for: Reconfigurable Optoelectronic Logic Gates Enabled by a VSe2/MoTe2 van der Waals Heterojunction
Source: Nanomaterials (Basel). 2026 Oct 2;16(19):1247. doi: 10.3390/nano16191247 (PMC13666891; doi:10.3390/nano16191247)
Supplement: Supplementary file 1 [file nanomaterials-16-01247-s001.zip › nanomaterials-4593547-supplementary.pdf]

Reconfigurable Optoelectronic Logic Gates Enabled by a VSe_2/$MoTe_2$ van der Waals Heterojunction

Kang Zhang [1], Wansong He [1], Ruya Cong [1], Xin Zhang [2], Jianzhi Gao [2], Yubing Tu [1,3,]*, Suofu Wang [4,]* and Mingsheng Long [1,3]

[1] State Key Laboratory of Opto-Electronic Information Acquisition and Protection Technology, Institutes of Physical Science and Information Technology, Anhui University, Hefei 230601, China
[2] School of Physics and Information Technology, Shaanxi Normal University, Xi'an 710119, China
[3] Key Laboratory of Opto-Electronic Information Acquisition and Manipulation of Ministry of Education, Anhui University, Hefei 230601, China
[4] School of Energy, Power and Safety, Anhui University of Applied Technology, Hefei 230011, China
* Correspondence: tuyubing@ahu.edu.cn (Y.T.); wangsuofu@uta.edu.cn (S.W.)

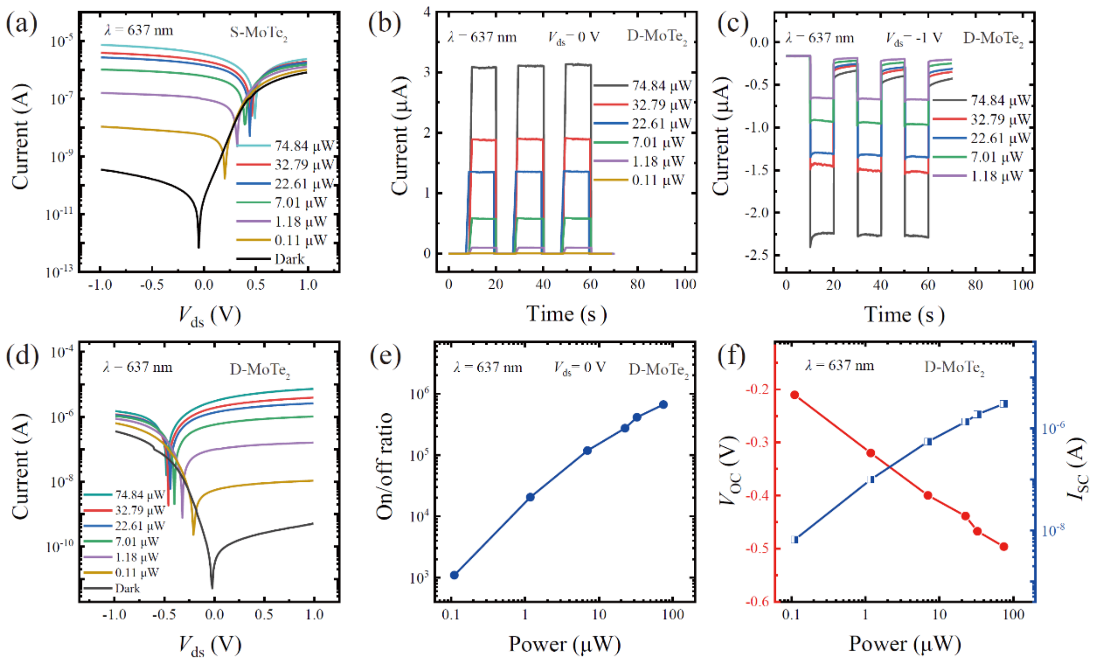

Figure S1. Photovoltaic characteristics of the VSe_2/$MoTe_2$ heterostructure device under 637 nm laser illumination. (a) *I*-*V* curves of the S-$MoTe_2$ configuration under different illumination powers. (b,c) Time-resolved photoresponse of the D-$MoTe_2$ configuration under different illumination powers at V_{ds} = 0 and −1 V, respectively. (d) *I*-*V* curves of the D-$MoTe_2$ configuration under different illumination powers. (e) On/off ratio as a function of illumination power for the D-$MoTe_2$ configuration at V_{ds} = 0 V. (f) Extracted V_{OC} and I_{SC} as a function of illumination power for the D-$MoTe_2$ configuration.

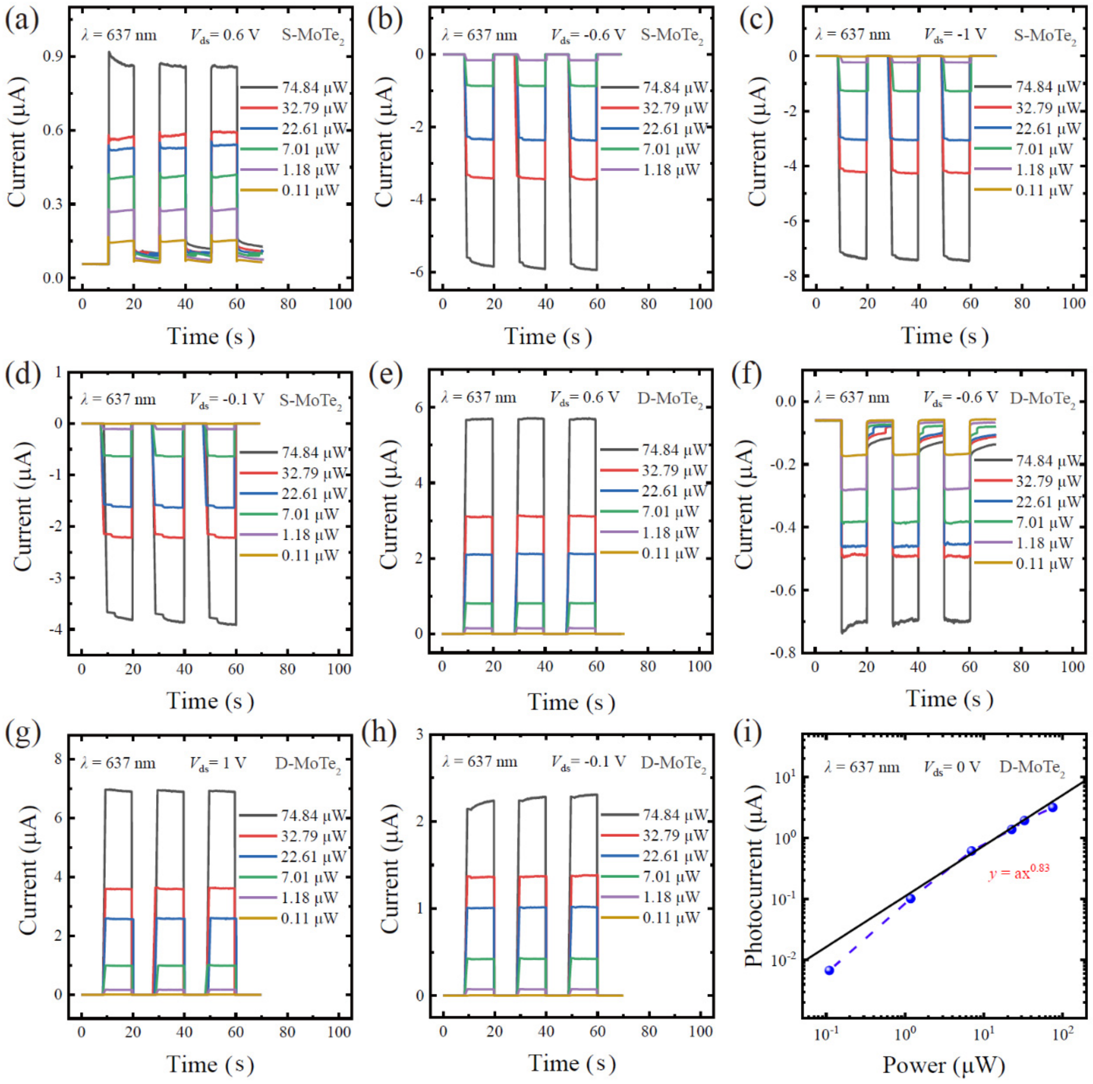

Figure S2. Photoresponse characteristics of the $VSe_2/MoTe_2$ heterostructure device at different bias voltages. (a–d) Time-resolved photoresponse of the S-$MoTe_2$ configuration under different illumination powers at 0.6V, -0.6V, -1V, and -0.1V, respectively. (e–h) Time-resolved photoresponse of the D-$MoTe_2$ configuration under different illumination powers at 0.6V, -0.6V, 1V, and -0.1V, respectively. (i) Photocurrent as a function of illumination power for the D-$MoTe_2$ configuration, with the black solid line showing the fit $y = ax^{0.83}$.

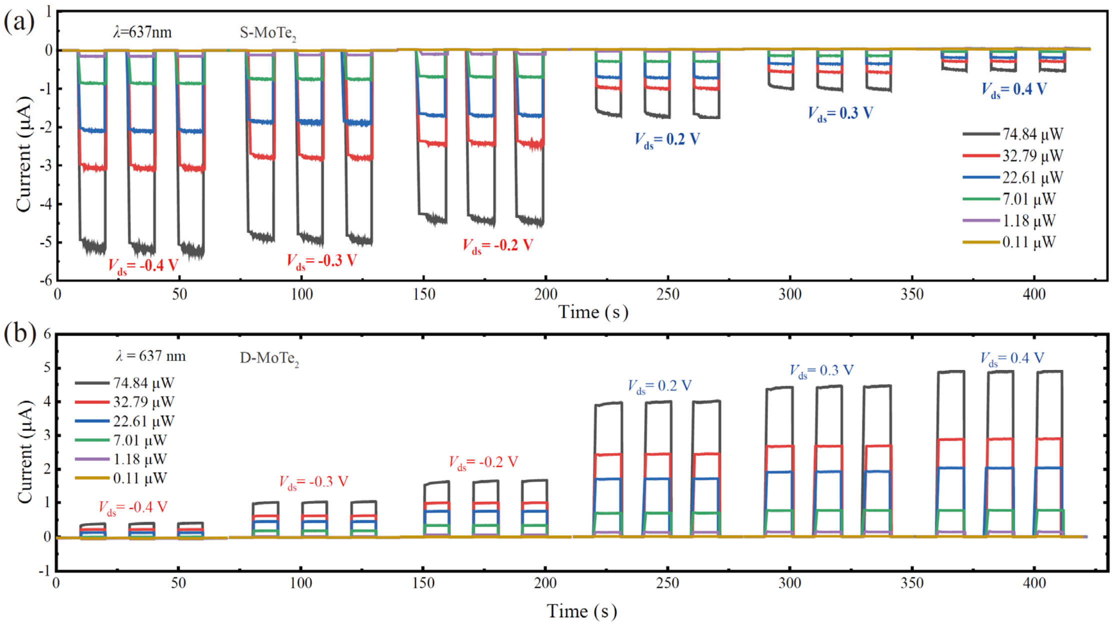

Figure S3. Bias-dependent photoresponse of the $VSe_2/MoTe_2$ heterostructure device under 637 nm laser illumination. (a, b) Time-resolved photoresponse of the S–$MoTe_2$ and D–$MoTe_2$ configurations, respectively, under different illumination powers and bias voltages.

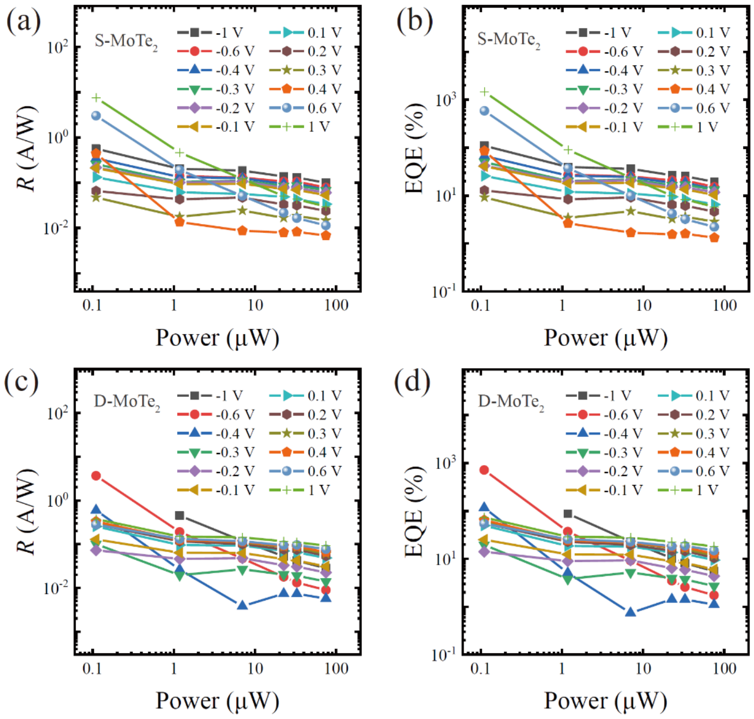

Figure S4. Power-dependent photodetection performance of the VSe_2/$MoTe_2$ heterostructure device. (a) and (b) *R* and EQE as a function of illumination power for the S-$MoTe_2$ configuration at different bias voltages, respectively. (c) and (d) *R* and EQE as a function of illumination power for the D-$MoTe_2$ configuration at different bias voltages, respectively.
